# Identification of Novel Risk Variants of Inflammatory Factors Related to Myeloproliferative Neoplasm: A Bidirectional Mendelian Randomization Study

**DOI:** 10.1055/s-0044-1779665

**Published:** 2024-02-12

**Authors:** Yang Li, Ting Sun, Jia Chen, Lei Zhang

**Affiliations:** 1 State Key Laboratory of Experimental Hematology, National Clinical Research Center for Blood Diseases, Haihe Laboratory of Cell Ecosystem, Institute of Hematology and Blood Diseases Hospital, Chinese Academy of Medical Sciences and Peking Union Medical College, Tianjin Key Laboratory of Gene Therapy for Blood Diseases, CAMS Key Laboratory of Gene Therapy for Blood Diseases, Tianjin, People's Republic of China; 2 Tianjin Institutes of Health Science, Tianjin, People's Republic of China; 3 School of Population Medicine and Public Health, Chinese Academy of Medical Sciences and Peking Union Medical College, Beijing, People's Republic of China

**Keywords:** myeloproliferative neoplasms, inflammatory regulators, bidirectional Mendelian randomization

## Abstract

Epidemiological and experimental evidence has linked chronic inflammation to the etiology of myeloproliferative neoplasm (MPN). However, it remains unclear whether genetic associations with specific inflammatory biomarkers are causal or due to bias. This study aimed to assess the effect of C-reactive protein (CRP) and systemic inflammatory regulators on MPN within a bidirectional Mendelian randomization design. Genetic associations with MPN were derived from a publicly available genome-wide association study (GWAS) comprising 1,086 cases and 407,155 controls of European ancestry. Additionally, data on inflammation were extracted from two GWASs focusing on CRP and cytokines. The causal relationships between exposure and outcome were explored using the inverse variance weighted (IVW) method. To confirm the final results, multiple sensitivity analyses, including MR-Egger, weighted median, and MR-pleiotropy residual sum and outlier (MR-PRESSO), were simultaneously employed. Our results suggest that lower levels of macrophage-migration inhibitory factor (IVW estimate odds ratio [OR IVW] per SD genetic cytokines change: 0.641; 95% confidence interval [CI]: 0.427–0.964; *p* = 0.032) and higher levels of interleukin-2 receptor α (lL2Rα, 1.377, 95% CI: 1.006–1.883; *p* = 0.046) are associated with an increased risk of MPN. Genetically predicted MPN is related to increased levels of RANTES (IVW estimate β: 0.043, 95% CI: 0.002–0.084; *p* = 0.039) and interleukin-10 (IVW estimate β: 0.030, 95% CI: 0.001–0.060; *p* = 0.041). This study provides evidence for a causal relationship between CRP, systemic inflammatory regulators, and MPN, and new insights into the etiology, prevention, and prognosis of MPN.

## Introduction

Myeloproliferative neoplasms (MPNs) are clonal hematopoietic disorders marked by the abnormal overproduction of differentiated hematopoietic cells during the chronic phase. The classical Ph-negative MPNs encompass polycythemia vera (PV), essential thrombocythemia (ET), and primary myelofibrosis (PMF). 1 Driver gene mutations, including JAK2, CALR, and MPL, play a crucial role in activating signaling pathways such as JAK-STAT. This activation leads to clonal hematopoiesis and the proliferation of specific lineages of bone marrow blood cells. Consequently, patients experience elevated white blood cell count, platelet levels, or red blood cell count, often accompanied by bleeding, thrombosis, and progressive splenomegaly. 2 The impact of disease-related complications and the elevated risk of progressing to acute myeloid leukemia (AML) significantly influence both the quality of life and overall survival (OS) of individuals within this patient population. The 10-year transformation rates from PV, ET, and PMF to AML are 2–4%, 1%, and 10–20%, respectively. 3 Previous studies have shown that aging leads to the continuous accumulation of DNA replication damage within hematopoietic stem cells, 4 as well as adaptive changes induced by external stresses such as radiation and chemotherapy, 5 bone marrow microenvironment defects and abnormalities, and germ line mutations, all of which can increase the risk of developing MPN and promote disease progression. 6 Nevertheless, the precise underlying causes of MPN are still not thoroughly comprehended.

In recent years, the role of inflammation in MPN has been increasingly recognized. Regardless of whether the patient has JAK2, MPL, and CALR mutations, the JAK/STAT signaling pathway is activated, leading to excessive proliferation of erythroid, granulocytic, and megakaryocytic cells and inducing the expression of inflammation-related genes. 6 Consequently, this activation results in the continuous release of inflammatory cytokines from granulocytes, thereby activating transcription factors such as nuclear factor kB and hypoxia-inducible factor-1α. 7 8 These transcription factors play a crucial role in regulating the expression of various inflammatory cytokines and the accumulation of reactive oxygen species, thus creating an inflammatory microenvironment within MPN. The released inflammatory cytokines further intensify the activation of JAK/STAT3 and nuclear factor kB signaling pathways through positive feedback loops, 9 progressively amplifying the inflammatory response. This, in turn, promotes the continuous proliferation of malignant clone cells in MPN and their acquisition of new mutations. Ultimately, this process can lead to the transformation of ET and PV into PMF and acute leukemia. 10 Multiple studies have demonstrated the close association of growth factors, interleukins, interferons, tumor necrosis factors, and chemokines with the progression of MPN. 11

Due to the potential limitations of observational studies, such as the inability to control for confounding factors and the presence of inverse causality, Mendelian randomization (MR) emerges as a more robust analytical method for making reliable etiological inferences. MR utilizes germ-line genetic variants as instrumental variables to estimate the long-term exposure to a particular factor of interest throughout an individual's lifetime. This approach offers a promising method to gain new insights into causal relationships between variables. 12 To investigate the potential association between systemic inflammatory factors and MPN, as well as to determine the direction of this association, a bidirectional MR study was conducted using the latest genome-wide association studies (GWASs) of systemic inflammatory regulators and MPN.

## Methods

### Study Design and MR Assumptions

**Fig. 1:**
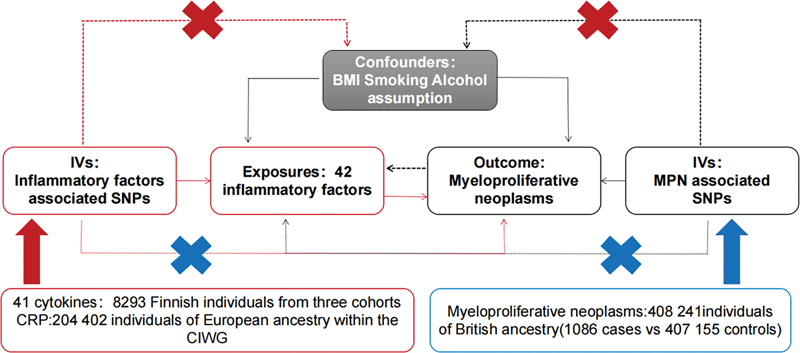
Assumptions and framework of the bidirectional Mendelian randomization (MR) investigation on the links between 42 inflammatory biomarkers and myeloproliferative neoplasm (MPN). The symbol ‘X’ denotes a non-permissible pathway. BMI, body mass index; CIWG, Cohorts for Inflammation Work Group; CRP, C-reactive protein; IVs, instrumental variables; SNPs, single nucleotide polymorphisms.

The study design is presented in Fig. 1 , illustrating a bidirectional MR approach to investigate the causal relationships between systemic inflammatory regulators and the risk of MPN. The methodology involved two sequential steps: first, the identification of genetic variants associated with each inflammatory factor to establish a causal association with MPN, and second, the identification of genetic variants associated with MPN to establish a causal relationship with the inflammatory factors.

MR is based on three fundamental assumptions: (1) the instrumental variables exhibit a strong association with the exposure variable; (2) the instrumental variables are independent of any confounding factors; and (3) the instrumental variables solely influence the outcomes through their impact on the exposure variable, without any involvement of alternative causal pathways. 13

### Genetic Associations with Systemic Inflammatory Regulators

The genetic variants associated with C-reactive protein (CRP) were obtained from two GWAS studies conducted on a dataset of 204,402 European individuals from 88 studies. Using an additive linear regression model, the association between genetic variants and CRP levels was evaluated at each study site. The model considered adjustments for age, sex, and population substructure, while also accounting for any relevant relatedness among individuals. 14

A study conducted on 8,293 Finnish individuals examined associations between genome variants and 41 cytokines and growth factors. The data utilized in this study were derived from the Cardiovascular Risk in Young Finns Study (YFS) and FINRISK surveys. To normalize the cytokine data, an inverse transformation was applied, and linear regression residuals were used to adjust for the first 10 genetic principal components. Additionally, a second inverse transformation was employed to ensure a normal distribution of the phenotypes. Effect sizes were reported in standard deviation units. 15

### Genetic Associations with MPN

Summary-level data on MPN were acquired from a GWAS of 408,241 individuals with European ancestry from the UK Biobank, which included 1,086 MPN cases and 407,155 controls. 16 Subsequently, a two-sample MR approach was utilized, incorporating the GWAS statistics to explore the causal connection between inflammatory factors and MPN. Notably, the samples for CRP, other inflammatory regulators, and MPN were sourced from separate consortia, ensuring no population overlap between the exposure and outcome groups.

### Selection of Genetic Instruments

To fulfill the assumptions of MR analysis ( Fig. 1 ), we exclusively incorporated single nucleotide polymorphisms (SNPs) that demonstrated robust and independent associations (R2 < 0.001 within 10,000 Kb) with the exposure variable, as reported in previously published GWASs, at a genome-wide significance level of *p* < 5 × 10 ^−8^ . Consequently, only six systemic inflammatory regulators and CRP met the criteria, possessing three or more independent SNPs. To expand the SNP pool, we applied less stringent thresholds to obtain additional SNPs. However, palindromic SNPs, which have the same alleles on both strands, are more difficult to harmonize compared with nonpalindromic SNPs. 13 Therefore, we excluded these SNPs from the analysis to prevent any ambiguity in the reported effect allele between the exposure and outcome GWAS. SNP potency was evaluated by calculating the F-statistic, which considers both the magnitude and precision of the genetic effect on the trait. The instrumental strength for the SNP-exposure association was determined by averaging the SNP-specific F-statistics, which were estimated as the square of the β divided by the variance for the SNP-exposure association. 17 SNPs with an F value below 10 were excluded, as a threshold above this value was deemed necessary to ensure the reliability of the SNPs. 18

### Statistical Analysis

To conduct our analysis, we required information on SNPs, including effect allele, other allele, effect sizes, standard errors, *p* -values, and allele frequencies. 19 In the main analysis, estimates were derived using inverse variance weighting (IVW) meta-analysis with multiplicative random effects, assuming balanced pleiotropy. This method provides a concise estimation and considers potential heterogeneity among SNP-based Wald ratio estimates. 20 Given the challenge in substantiating the “exclusion-restriction” hypothesis, various sensitivity analyses were conducted to explore different assumptions, encompassing techniques such as MR Egger, weighted median (WM), and MR Pleiotropy RESidual Sum and Outlier (MR-PRESSO), to validate the findings. Taking into account the array of systemic inflammatory regulators examined, we implemented a Bonferroni adjustment to account for multiple comparisons. Findings that displayed significance prior to correction but not afterward were regarded as indicative. For the primary analyses, power calculations were conducted using an approximation that considers the sample size for exposure on outcome divided by the *r* ^2^ for instruments on exposure, as suggested by previous research. 21

### Sensitivity Analysis

Sensitivity analysis is crucial in MR studies, as it helps to identify underlying pleiotropy, and highlights that heterogeneity can severely compromise MR estimates. To represent potential horizontal pleiotropy, we utilized heterogeneity markers identified through Cochran's Q test ( *p* < 0.05) using the IVW approach. The MR-Egger regression yielded an intercept that functioned as an indicative factor for identifying directional pleiotropy, with a significance threshold set at a *p* -value of less than 0.05 to ascertain its existence. Nevertheless, our primary emphasis was placed on the consistency of estimate direction rather than the statistical significance of the estimates, considering the limited statistical power associated with MR-Egger. MR-PRESSO employs a comprehensive examination to identify horizontal pleiotropy, and in cases where needed, it can address potential pleiotropic outliers by means of removing those outliers.

The MR analyses were executed within the R environment (version 3.4.2) utilizing the R packages “TwosampleMR” and “MR-PRESSO.” Since publicly accessible summary data were utilized, ethical approval was not necessary.

## Results

### The Causal Effect of Genetically Determined CRP on the Risk of MPN

**Fig. 2:**
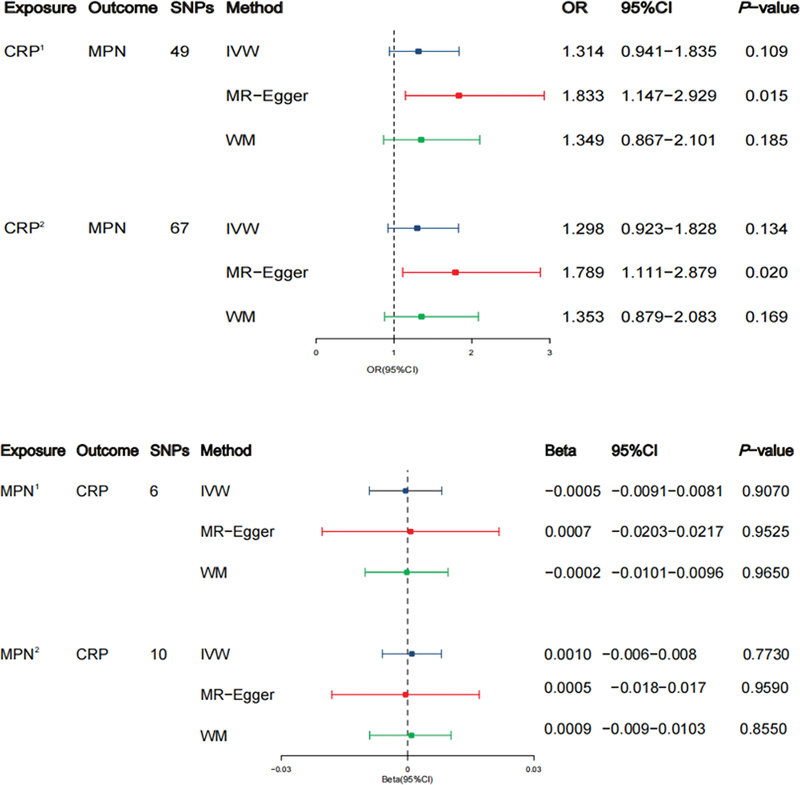
Mutual causal influences of C-reactive protein (CRP) and myeloproliferative neoplasm (MPN). CRP1 indicates that the single nucleotide polymorphisms (SNPs) are associated with exposure at a genome-wide significance level. CRP2 indicates that the SNPs are associated with exposure at *p* < 5 × 10 ^−7^ . MPN1 indicates that the SNPs are associated with exposure at *p* < 5 × 10 ^−6^ . MPN2 indicates that the SNPs are associated with exposure at *p* < 1 × 10 ^−5^ . IVW, inverse variance weighting; WM, weighted median.

We identified a set of 49 SNPs across the genome displaying a significant correlation with CRP ( Supplementary Table S1 [online only]), as well as 67 SNPs associated with CRP at a less strict threshold ( *p* < 5 × 10 ^−7^ , Supplementary Table S2 [online only]). Employing the IVW method, we detected no indications of an association between genetically predicted CRP levels and MPN risk, whether employing SNPs of genome-wide significance or those with a less stringent criterion ( *p* -values of 0.109 and 0.134, respectively, Fig. 2 ). Findings from the WM method aligned with those obtained through the IVW approach. However, when utilizing the MR-Egger method, we observed a significant link between genetically predicted CRP levels and MPN risk, using both SNPs of genome-wide significance and those with a less strict threshold ( *p* -values of 0.015 and 0.020, respectively, Fig. 2 ). The final outcome was derived from the IVW method.

### The Causal Effect of Genetically Determined MPN on CRP Level

We identified 6 SNPs associated with MPN using a less stringent criterion of *p* < 5 × 10 ^−6^ ( Supplementary Table S3 [online only]), and an additional 10 SNPs using a cutoff of *p* < 1 × 10 ^−5^ ( Supplementary Table S4 [online only]). Our analysis revealed no substantial evidence supporting a causal effect of MPN on CRP using either the *p* < 5 × 10 ^−6^ cutoff ( *p* = 0.907, Fig. 2 ) or the *p* < 1 × 10 ^−5^ cutoff ( *p* = 0.773, Fig. 2 ). The findings from other MR methods were in line with those obtained through the IVW method ( Fig. 2 ), and no indications of horizontal pleiotropy were detected ( Supplementary Table S5 [online only]).

### The Causal Effect of Systemic Inflammatory Regulators on Risk of MPN

**Fig. 3:**
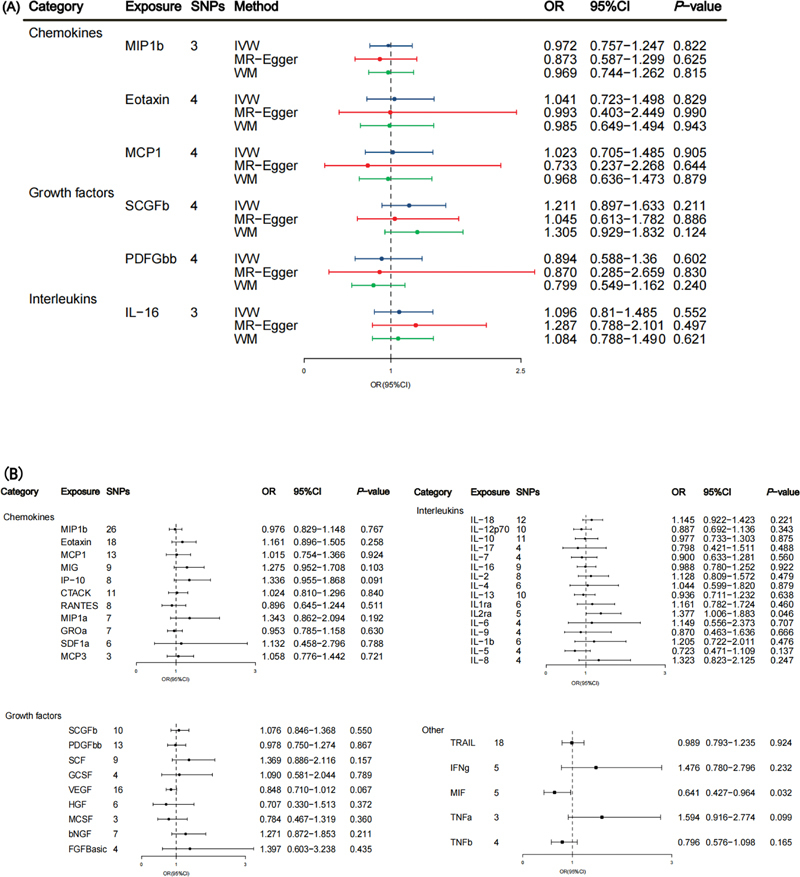
**(A)** Associations between genetically predicted systemic inflammatory regulators and MPN with genome-wide significant SNPs. **(B)** Associations between genetically predicted systemic inflammatory regulators and MPN with SNPs reaching *p* < 5 × 10 ^−6^ . bNGF, β nerve growth factor; CTACK, cutaneous T cell attracting chemokine; FGFBasic, basic fibroblast growth factor; GCSF, granulocyte colony-stimulating factor; GROa, growth-regulated oncogene-a; HGF, hepatocyte growth factor; IFNg, interferon gamma; IL, interleukin; IP, interferon-gamma-induced protein 10; MCP1, monocyte chemotactic protein-1; MCP3, monocyte-specific chemokine 3; MCSF, macrophage colony-stimulating factor; MIF, macrophage-migration inhibitory factor; MIG, monokine induced by interferon gamma; MIP1a, macrophage inflammatory protein-1a; MIP1b, macrophage inflammatory protein-1b; PDGFbb, platelet-derived growth factor BB; RANTES, regulated on Activation, Normal T Cell Expressed and Secreted; SCF, stem cell factor; SCGFb, stem cell growth factor β; SDF1a, stromal cell-derived factor-1 α; SNPs, single-nucleotide polymorphisms; TNFa, tumor necrosis factor α; TNFb, tumor necrosis factor β; TRAIL, TNF-related apoptosis-inducing ligand; VEGF, vascular endothelial growth factor.

The F-statistics of the genome-wide significant SNPs related to the six inflammatory regulators (MIP1b, Eotaxin, MCP 1, SCGFb, PDFGbb, and interleukin-16 [IL-16]) ranged from 30.83 to 636.49, denoting their robustness as instrumental variables ( Supplementary Table S6 [online only]). The impacts of these six systemic inflammatory regulators, as predicted by the genome-wide significant SNPs, on MPN risk are presented in Fig. 3A . Despite accounting for multiple comparisons, our analyses did not uncover any significant associations between these inflammatory regulators and MPN. Furthermore, the MR-Egger intercept test failed to identify any horizontal pleiotropy, while the Cochran Q tests indicated no signs of heterogeneity ( Supplementary Table S7 [online only]).

We implemented a less stringent criterion ( *p* < 5 × 10 ^−6^ ) to incorporate all 41 inflammatory regulators, characterized by F-statistics ranging from 11.16 to 784 ( Supplementary Table S8 [online only]). Employing the IVW method, we identified IL2Rα and macrophage-migration inhibitory factor (MIF) as potential causal factors associated with MPN. However, sensitivity analyses yielded inconsistent estimates ( Fig. 3B , Supplementary Table S9 ). While no significant effects were observed using the WM or MR-Egger methods, the direction of the effect aligned with the IVW method for both ( Supplementary Table S9 [online only]). Notably, we found no significant indications of horizontal pleiotropy based on the MR-Egger intercept test, and MR-PRESSO analysis did not identify any outliers ( Supplementary Table S9 [online only]).

**Fig. 4:**
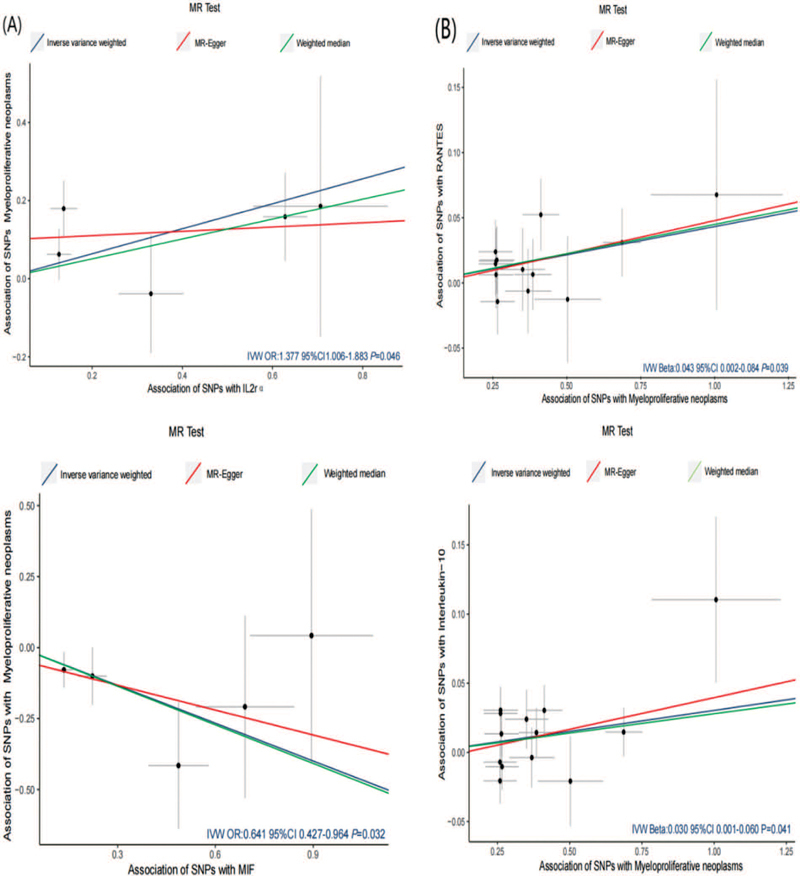
**(A)** The Mendelian randomization (MR) scatter plots illustrating the relationship between interleukin receptor (ILR) 2α (IL2Rα) and macrophage migration inhibitory factor (MIF) in myeloproliferative neoplasm (MPN). **(B)** MR scatter plots illustrating the relationship between myeloproliferative neoplasm (MPN) and RANTES and interleukin 10 (IL-10). Individual inverse variance (IV) associations with cytokine risk are displayed versus individual IV associations with MPN in black dots. The 95% CI of odd ratio for each IV is shown by vertical and horizontal lines. The slope of the lines represents the estimated causal effect of the MR methods. CI, confidence interval.

As depicted in Fig. 3B , IL2Rα (odds ratio [OR] IVW: 1.377; 95% confidence interval [CI]: 1.006–1.883; *p* = 0.046) exhibited suggestive evidence of being associated with an increased risk of MPN, while MIF demonstrated a negative association with MPN risk (OR IVW: 0.641; 95% CI: 0.427–0.964; *p* = 0.032). Fig. 4A provides the MR scatter plot illustrating the relationship between IL2Rα, MIF, and MPN.

### The Causal Effect of MPN on Systemic Inflammatory Regulator Levels

**Fig. 5:**
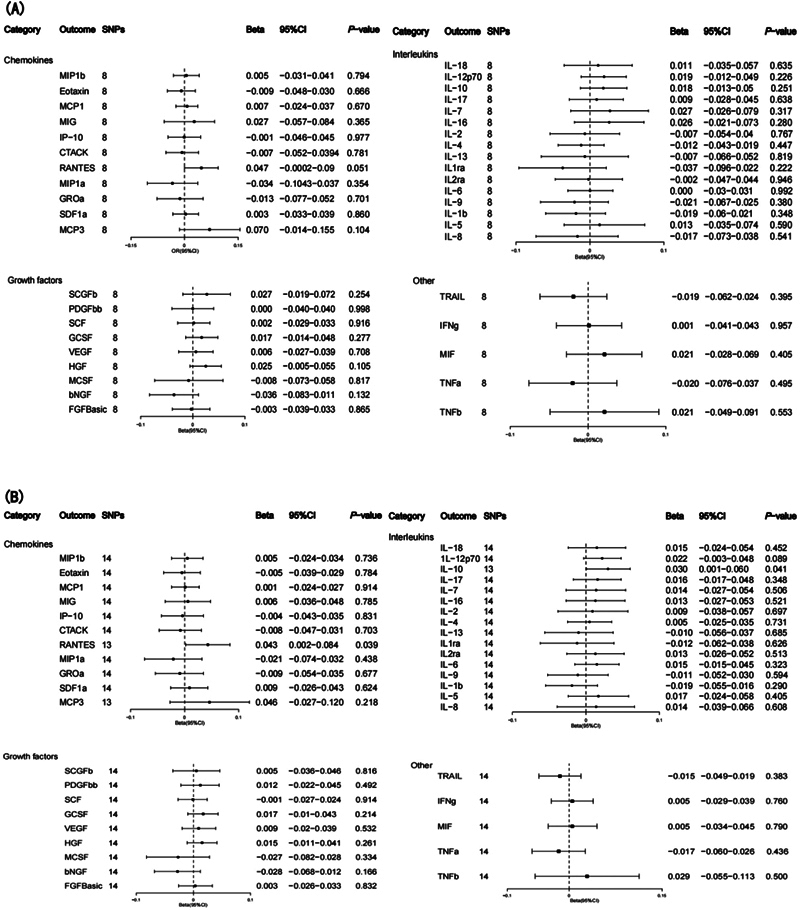
**(A)** Associations between genetically predicted MPN and systemic inflammatory regulators at *p* < 5 × 10 ^−6^ . **(B)** Associations between genetically predicted MPN and systemic inflammatory regulators at *p* < 1 × 10 ^−5^ . bNGF, β nerve growth factor; CTACK, cutaneous T cell attracting chemokine; FGFBasic, basic fibroblast growth factor; GCSF, granulocyte colony-stimulating factor; GROa, growth-regulated oncogene-a; HGF, hepatocyte growth factor; IFNg, interferon gamma; IL, interleukin; IP, interferon-gamma-induced protein 10; MCP1, monocyte chemotactic protein-1; MCP3, monocyte-specific chemokine 3; MCSF, macrophage colony-stimulating factor; MIF, macrophage-migration inhibitory factor; MIG, monokine induced by interferon gamma; MIP1a, macrophage inflammatory protein-1a; MIP1b, macrophage inflammatory protein-1b; PDGFbb, platelet-derived growth factor BB; RANTES, regulated on Activation, Normal T Cell Expressed and Secreted; SCF, stem cell factor; SCGFb, stem cell growth factor β; SDF1a, stromal cell-derived factor-1 α; SNPs, single-nucleotide polymorphisms; TNFa, tumor necrosis factor α; TNFb, tumor necrosis factor β; TRAIL, TNF-related apoptosis-inducing ligand; VEGF, vascular endothelial growth factor.

To investigate the potential effects of reverse causation, we carefully selected 11 SNPs associated with MPN using a cutoff of *p* < 5 × 10 ^−6^ . However, due to their palindromic nature and intermediate allele frequency, three of these SNPs (rs12435976, rs144317085, and rs7545069) were excluded from further analysis. As a result, we extracted a set of eight SNPs that exhibited strong and independent associations with MPN at a significance level of *p* < 5 × 10 ^−6^ ( Supplementary Table S10 [online only]). Utilizing the IVW method, we discovered suggestive evidence indicating that a genetically higher risk of MPN might be associated with increased RANTES levels (β IVW = 0.047, 95% CI: [−0.0003, 0.094], *p* = 0.051; Fig. 5A ). No other significant associations were observed. The MR-Egger and WM methods both indicated a null causal effect of MPN risk on RANTES concentration (β MR-Egger = 0.051, 95% CI: [−0.085, 0.187], *p* = 0.49; Supplementary Table S11 [online only]; β WM = 0.042, 95% CI: [−0.017, 0.102], *p* = 0.163; Supplementary Table S11 [online only]).

As previously mentioned, we included only eight SNPs in our analysis, which limits our ability to draw definitive conclusions. To explore additional possibilities and enhance the robustness of our findings, we adopted a more lenient criterion and included 14 SNPs associated with MPN using a less stringent cut-off ( *p* < 1 × 10 ^−5^ ). The F-statistics for these SNPs ranged from 19.60 to 117.72 ( Supplementary Table S12 ). However, due to the unavailability of certain SNPs for cytokines, even after employing proxy SNPs and the harmonization step, the number of SNPs varied for different cytokines ( Supplementary Table S12 [online only]).

Applying the IVW method, we found suggestive associations indicating that a genetically higher risk of MPN might be linked to increased levels of RANTES (β IVW = 0.043, 95% CI: [0.002, 0.084], *p* = 0.039; Fig. 5B ) and IL-10 (β IVW = 0.030, 95% CI: [0.001, 0.060], *p* = 0.041; Fig. 5B ). No other significant associations were observed. The WM and MR-Egger methods also demonstrated null causal effects of MPN risk on RANTES and IL-10 concentration ( Supplementary Table S13 [online only]). The MR scatter plots and funnel plots illustrating the relationship between MPN and RANTES, as well as IL-10, can be found in Fig. 4B and Supplementary Fig. S1 (online only).

## Discussion

In our study, we utilized the latest GWAS data to perform a bidirectional MR analysis to evaluate the potential causal association between CRP and systemic inflammatory regulators with MPN. In this bidirectional MR analysis, we found that higher genetically determined IL2Rα was associated with increased risk of MPN, while higher genetically determined MIF may decrease risk of MPN. Conversely, our results also indicated that MPN was causally associated with increased levels of RANTES and IL-10, suggesting a potential proinflammatory effect of MPN. However, we did not find any indications of a causal association between CRP, as well as other inflammatory regulators, and MPN. These findings propose IL2Rα and MIF as potential targets for therapeutic interventions in MPN. The results of our study support the notion that inflammatory factors have an impact on the progression of MPN.

The causal relationship between inflammation and MPN malignant clones is not yet fully understood. Currently, there are two pathways believed to promote tumor formation: the exogenous pathway, in which inflammation increases the risk of tumor formation, and the endogenous pathway, in which driver gene mutations such as JAK2, MPL, and CALR lead to the formation of an inflammatory microenvironment. 2

The JAK2V617F mutation mainly activates STAT3 and STAT5, mediating the transmission of erythropoietin, thrombopoietin, and granulocyte colony-stimulating factor signaling pathways, resulting in excessive proliferation of cells in the erythroid, granulocytic, and megakaryocytic lineages, and the production of inflammatory factors. 22 The levels of IL-6, IL-9, C–C motif chemokine ligand (CCL)3, CCL4, CCL11, CXC motif chemokine ligand (CXCL)5, CXCL9, and CXCL10 in the serum of 6-month-old mice carrying the JAK2V617F mutation were found to be significantly elevated compared with those in the wild-type control mice. 23 Additionally, in the serum of mice with an MPL W515L overexpression myelofibrosis model, the concentrations of TNF-α, CCL2, CXCL9, IL-6, CXCL10, and IL-12 were notably elevated compared with the levels observed in wild-type control mice. 24 A study conducted by Tefferi et al 25 demonstrated that elevated levels of plasma IL-8, IL-2R, IL-12, IL-15, and interferon-gamma-inducible protein (IP)-10 were significantly associated with a reduced OS period in 90 PMF patients. Similarly, Øbro et al 26 conducted another investigation that identified significant abnormalities in the expression of epidermal growth factor, CCL11, growth regulated oncogene (GRO)-α, TGF-α, IL-1Rα, TNF-α, IL-6, IL-8, IP-10, and interferon-gamma in at least 185 patients with an MPN subtype. Notably, elevated levels of IL-6 were significantly correlated with a shorter OS. The JAK1/JAK2 kinase inhibitor ruxolitinib can inhibit the activity of the JAK/STAT signaling pathway. Clinical trial results showed that 23 MF patients who received ruxolitinib treatment had varying degrees of decreased levels of plasma inflammatory factors IL-6, TNF-α, MIP-lB, and IL-1R. 27 Highly investigated markers of inflammation in MPN include high-sensitivity CRP (hs-CRP) and pentraxin-3 (PTX-3). Barbu et al 28 conducted a study in 2011 revealing a correlation between the expression levels of PTX-3 and hs-CRP and a JAK2V617F allele burden exceeding 50%. In MPN patients, elevated levels of hs-CRP (>3 mg/L) are associated with an increased risk of thrombotic events, whereas higher levels of PTX-3 (>4.5 ng/mL) are linked to a reduced risk of thrombosis. However, the divergent conclusions obtained from the literature may be attributed to different patient groups with different stages of the disease, as well as the heterogeneity of MPN-related treatments.

In our comprehensive MR investigation, we effectively discovered two primary regulators and two consequential factors associated with MPN. Previous studies have shown that MIF is a trimeric protein that acts as a pluripotent cytokine with proinflammatory properties. While initially associated with activated T cells, MIF has since been acknowledged as a versatile cytokine secreted by diverse cell types participating in immune responses and physiological processes. 29 There is rapidly growing evidence showing that MIF plays a positive regulatory role in macrophage responses during infection by enhancing the production of pro-inflammatory cytokines, such as TNF-α, IFN-γ, IL-1β, IL-2, IL-6, and IL-8, as well as other inflammatory components like nitric oxide and COX2, ultimately aiding in the clearance of pathogens. 30 However, our findings suggest that the increased genetic susceptibility to MIF may be related to the reduced risk of MPN. Additionally, recent studies have shown that highly expressed MIF gene variants significantly reduce susceptibility to a few autoimmune diseases such as systemic lupus erythematosus and psoriasis. 31 32 Currently, the role of MIF in the progression of MPN disease remains unexplored. However, utilizing the MR method and data from a GWAS, we have unveiled a remarkable and previously unrecognized negative causal relationship between MIF and MPN. We intend to validate this conclusion through animal experiments in the future. The composition of IL-2R, a functional receptor, consists of three crucial constituents: IL2Rα, which is alternatively referred to as CD25 and serves as the α chain; IL2Rβ, also known as CD122, functioning as the β chain; and IL2Rγ, known as CD132, acting as the γ chain. 33 In humans, the expression of IL2Rα demonstrates significant elevation in a diverse array of hematological malignancies, while its presence is limited to only a handful of solid tumor types. Notable solid tumors exhibiting heightened sCD25 levels encompass lung adenocarcinoma, esophageal cancer, and head and neck cancer. 33 In addition, hematological malignancies displaying elevated sCD25 concentrations include select cases of acute leukemias and non-Hodgkin's lymphoma. 34 The current understanding identifies the cell surface interleukin-2 receptor α-chain (IL-2Rα, CD25) as a robust prognostic indicator for unfavorable outcomes in patients diagnosed with AML. 35 The treatment of tumors targeting IL2Rα has seen the development of two distinct approaches. The initial strategy encompasses the utilization of immunomodulators, such as recombinant IL-2, mutant IL-2, and monoclonal antibodies against IL2Rα. These agents effectively influence the initiation and progression of tumors. Conversely, the second approach focuses on selectively delivering cytotoxins to IL2Rα+ cells, which encompass both tumor cells and immune cells responsible for regulating tumor cell survival. 36

In the opposite direction, we also discovered that MPN causes elevated RANTES and IL-10 levels. RANTES, also known as CCL5 (Regulated upon Activation, Normal T Cell Expressed and Presumably Secreted), is categorized within the CC subfamily of chemokines. Various inflammatory cells are capable of expressing CCL5, with T cells and monocytes being the predominant cell types in terms of CCL5 expression. Among the receptors it can bind to, CCL5 exhibits the strongest affinity for CCR5, along with its ability to bind to CCR1, CCR3, and CCR4. 37 Recent research has extensively investigated the role of the CCL5/CCR5 axis in tumorigenesis across multiple cancer types, including chondrosarcoma, gastric cancer, breast cancer, pancreatic cancer, and head and neck cancer. 38 Furthermore, this axis plays a significant role in promoting tumor progression in hematological cancers such as acute lymphoblastic leukemia, AML, chronic myeloid leukemia, 39 classic Hodgkin lymphoma, 40 and multiple myeloma. 41 Creating a more favorable microenvironment for the survival of tumor cells is a primary objective of the CCL5/CCR5 axis. This axis facilitates cell signaling pathways such as PI3K/AKT, NF-kB, ERK/MEK, and HIF-a, leading to uncontrolled proliferation and immortality of tumor cells. 42 Simultaneously, these signaling pathways regulate MMPs, growth factors, and inflammatory factors, thus removing obstacles for tumor invasion and metastasis. Additionally, the recruitment of Tregs, MDSCs, and TAM by CCL5/CCR5 contributes to the induction of tumor immunosuppression. 43 IL-10, a cytokine with pleiotropic immunosuppressive functions, was the first member of the IL-10 family of cytokines to be identified. Previous basic research has found that in the MPL W515L overexpressing MF mouse model serum, the level of IL-10 was significantly higher than that in wild-type control mice. Additionally, the CALR mutation can stimulate monocytes to produce excessive levels of IL-10. 8 24 The bidirectional regulation of IL-10 makes its role unclear in different studies.

In this study, we have incorporated several methodological strengths worth highlighting. First, we accessed the most up-to-date and comprehensive dataset available on cytokine levels, along with the largest GWAS dataset for MPN. Additionally, our findings underwent thorough validation through sensitivity analyses for pleiotropy, utilizing alternative MR methods. This unique approach holds significant importance in uncovering whether a genetic predisposition to MPN can induce alterations in circulating inflammatory factors and whether the risk of MPN can be influenced by high or low levels of these factors. Notably, this particular relationship has not been previously explored in MR studies. Lastly, our study ventured beyond the commonly employed genome-wide significance *p* -value threshold of 5 × 10 ^−8^ by employing less stringent cutoffs (1 × 10 ^−5^ and 5 × 10 ^−6^ ) for lower frequency variants in individuals of European ancestry, thus uncovering additional possibilities.

Furthermore, it is essential to recognize several limitations present in our study. First, the patient cohort consisted exclusively of individuals with European ancestry, limiting the ability to establish a causal relationship between inflammatory factors and MPN in diverse populations. Moreover, the intricate nature of MPN, encompassing varied etiology and prognosis within each clinical symptom, was not extensively explored. In addition, unpredictable factors in real-world clinical scenarios may affect changes in inflammatory factors, and outcome misclassification within the UK Biobank could introduce bias, particularly for individuals without medical records due to loss to follow-up or emigration from the United Kingdom.

In summary, our comprehensive MR analysis unveiled potential associations between a set of systemic inflammatory regulators and the onset of MPN, as well as their causal influence on specific MPN types. However, to unravel the underlying mechanisms governing the role of systemic inflammatory regulators in MPN, additional in-depth investigations are required in the future. Nonetheless, this study contributes valuable insights into the intricate connections between systemic inflammation and MPN, potentially paving the way for novel insights into the etiology and the discovery of new biomarkers for the diagnosis and treatment of MPN in clinical settings.
