# Supplementary material for: Identification of Novel Risk Variants of Inflammatory Factors Related to Myeloproliferative Neoplasm: A Bidirectional Mendelian Randomization Study
Source: Glob Med Genet. 2024 Feb 12;11(1):48–58. doi: 10.1055/s-0044-1779665 (PMC10861317; doi:10.1055/s-0044-1779665)
Supplement: Supplementary file 1 — Supplementary Material [file 10-1055-s-0044-1779665-s2300084.pdf]

Supplementary Table S1 Details of C-reactive protein predicting SNPs with myeloproliferative neoplasm (with genome-wide significant SNPs)

C-reactive protein						Myeloproliferative neoplasm		*F*-statistics
SNP	Effect allele	Other allele	*p*-Value	Beta	Standard error	Beta	Standard error	
rs10512597	C	T	4.44E-14	0.036931	0.00489	0.1053	0.0669	57.03801678
rs1051338	G	T	2.27E-09	0.023881	0.003992	−0.0935	0.0591	35.78688947
rs10521222	T	C	2.06E-22	−0.104411	0.010714	−0.1498	0.1805	94.97060977
rs10832027	A	G	4.43E-12	0.025944	0.003745	−0.0954	0.0568	47.99215231
rs10925027	C	T	4.25E-21	−0.036035	0.00382	0.0073	0.058	88.98613148
rs12202641	T	C	3.00E-10	−0.022804	0.003617	0.0414	0.0578	39.74889382
rs12587622	A	G	8.52E-09	−0.020798	0.003609	−0.0231	0.0564	33.21003885
rs1260326	C	T	2.72E-92	−0.073462	0.003604	0.0102	0.0594	415.4855565
rs12960928	C	T	1.91E-09	0.024	0.003993	0.13	0.071	36.12633152
rs12995480	C	T	1.24E-10	0.031261	0.004855	−0.0895	0.0761	41.45980588
rs13233571	T	C	2.95E-25	−0.056895	0.005476	−0.0259	0.0846	107.9496714
rs13409371	A	G	5.07E-36	0.048232	0.003847	−0.0088	0.0622	157.1904543
rs1441169	G	A	2.27E-11	−0.024926	0.003725	0.0525	0.0575	44.77675607
rs1490384	T	C	2.65E-12	−0.024816	0.003545	0.0084	0.0564	49.0039493
rs1582763	A	G	2.37E-09	−0.022107	0.0037	−0.0534	0.0632	35.69901015
rs17658229	C	T	5.50E-09	0.055568	0.009522	0.0493	0.1319	34.05596397
rs178810	T	C	2.95E-08	0.02001	0.003606	−0.0171	0.0578	30.79234277
rs1800961	T	C	4.63E-23	−0.1115	0.011267	0.0433	0.1356	97.93392175
rs1805096	A	G	2.17E-183	−0.104381	0.003614	0.0395	0.0567	834.1931321
rs1880241	G	A	8.41E-14	−0.027537	0.003687	0.0105	0.058	55.78108711
rs2064009	T	C	2.28E-14	0.027111	0.003549	0.0455	0.0566	58.35513462
rs2239222	G	A	9.87E-20	0.035484	0.003901	0.0921	0.0587	82.73956638
rs2315008	G	T	5.36E-10	0.023467	0.003777	−0.0274	0.064	38.60301068
rs2352975	C	T	6.43E-10	0.024897	0.004026	−2.00E-04	0.065	38.24251956
rs2710804	C	T	1.30E-08	0.021262	0.003737	−0.0541	0.059	32.37144098
rs2794520	T	C	1.00E-200	−0.182186	0.003712	−0.151	0.0587	2408.873902
rs2836878	A	G	7.71E-26	−0.042902	0.004079	0.1263	0.0654	110.6235692
rs2852151	A	G	1.36E-11	0.024735	0.003655	−0.0019	0.0569	45.79826933
rs2891677	T	C	1.59E-08	0.019859	0.003511	−0.0704	0.0566	31.992862
rs3122633	C	T	1.68E-12	0.027479	0.00389	−0.0013	0.0599	49.90024128
rs3134899	T	C	4.93E-08	0.023329	0.004274	−0.1046	0.0814	29.79361563
rs340005	A	G	1.01E-15	0.030007	0.003736	−0.1722	0.0629	64.51065054
rs387976	C	A	1.05E-10	0.025507	0.003946	−0.0563	0.0623	41.78347947
rs4092465	G	A	3.11E-10	0.027483	0.004364	0.0557	0.0589	39.66055542
rs4129267	T	C	1.20E-129	−0.087519	0.003612	−0.0273	0.0618	587.0961199
rs4246598	A	C	5.11E-10	0.022063	0.003547	−0.0026	0.0582	38.69071478
rs4420638	G	A	1.00E-200	−0.229459	0.006122	−0.063	0.0642	1404.829255
rs4655802	A	G	1.88E-09	−0.025012	0.00416	−0.1152	0.0581	36.15015625
rs4656849	G	A	4.91E-54	0.057656	0.003723	0.0095	0.0611	239.8296898
rs469772	T	C	5.54E-12	−0.031327	0.004542	−0.0108	0.0693	47.57111759

 © 2024. The Author(s).

Supplementary Table S1 (*Continued*)

C-reactive protein						Myeloproliferative neoplasm		F-statistics
SNP	Effect allele	Other allele	p-Value	Beta	Standard error	Beta	Standard error	
rs4767920	A	G	4.00E-15	−0.038519	0.0049	−0.0014	0.0784	61.79564186
rs4841132	G	A	2.00E-25	0.065095	0.006243	0.0614	0.0815	108.719787
rs6001193	G	A	6.53E-14	−0.027809	0.003706	−0.1456	0.0589	56.30667914
rs644234	G	T	1.13E-09	0.022597	0.003708	−0.0957	0.0569	37.13830857
rs6601302	G	T	9.80E-12	−0.030518	0.004478	−0.0887	0.0646	46.44553383
rs6672627	A	C	2.89E-13	−0.037135	0.005083	−0.0129	0.0738	53.37361727
rs7121935	A	G	5.28E-09	−0.021853	0.00374	0.13	0.0596	34.14121143
rs7310409	G	A	1.00E-200	0.137075	0.003706	0.0738	0.0577	1368.061683
rs9284725	A	C	7.34E-11	−0.02731	0.00419	0.0734	0.0841	42.4830173

Note: F-statistic >10 suggests sufficient strength to ensure the validity of the instrumental variable. Beta for CRP represents change in percent per 1 copy of effect allele.

Supplementary Table S2 Details of C-reactive protein predicting SNPs with myeloproliferative neoplasm (with SNPs reaching $p < 5 \times 10^{-7}$)

►Table S2 displayed in Table S2.xlsx

Supplementary Table S3 Details of myeloproliferative neoplasm predicting SNPs with C-reactive protein (with SNPs reaching $p < 5 \times 10^{-6}$)

Myeloproliferative neoplasm						C-reactive protein		F-statistics
SNP	Effect allele	Other allele	p-Value	Beta	Standard error	Beta	Standard error	
rs10815145	A	G	2.00E-27	0.6868	0.0633	0.000512	0.004008	117.7207859
rs1260073	A	G	3.37E-06	−0.2658	0.0572	0.001955	0.003637	21.59324417
rs17039205	G	A	2.06E-06	0.3689	0.0777	0.001145	0.005948	22.54110868
rs458740	T	C	4.93E-06	0.259	0.0567	−0.00116	0.00372	20.86572169
rs4628318	A	G	4.78E-06	0.2589	0.0566	0.004645	0.004021	20.9233509
rs4726704	A	C	2.23E-06	−0.3506	0.0741	0.00615	0.005413	22.38656227

Abbreviation: SNP, single nucleotide polymorphism.
Note: F-statistic >10 suggests sufficient strength to ensure the validity of the instrumental variable. Beta for CRP represents change in percent per 1 copy of effect allele.

 © 2024. The Author(s).

Supplementary Table S4 Details of myeloproliferative neoplasm predicting SNPs with C-reactive protein (with SNPs reaching $p < 1 \times 10^{-5}$)

Myeloproliferative neoplasm						C-reactive protein		*F*-statistics
SNP	Effect allele	Other allele	*p*-Value	Beta	Standard error	Beta	Standard error	
rs10472076	C	T	6.37E-06	0.2636	0.0584	0.003393	0.004243	20.37347532
rs10815145	A	G	2.00E-27	0.6868	0.0633	0.001098	0.004022	117.7207859
rs1260073	A	G	3.37E-06	−0.2658	0.0572	0.001955	0.003637	21.59324417
rs13118899	A	G	6.29E-06	−0.2606	0.0577	−0.000688	0.003933	20.39845132
rs17039205	G	A	2.06E-06	0.3689	0.0777	0.001145	0.005948	22.54110868
rs458740	T	C	4.93E-06	0.259	0.0567	−0.00116	0.00372	20.86572169
rs4628318	A	G	4.78E-06	0.2589	0.0566	0.004645	0.004021	20.9233509
rs4726704	A	C	2.23E-06	−0.3506	0.0741	0.00615	0.005413	22.38656227
rs479784	A	G	9.54E-06	−0.3843	0.0868	−0.002873	0.005658	19.60204214
rs7608211	T	C	6.02E-06	1.0065	0.2224	−0.005914	0.011965	20.48132234

Abbreviation: SNP, single nucleotide polymorphism.
Note: F-statistic >10 suggests sufficient strength to ensure the validity of the instrumental variable. Beta for CRP represents change in percent per 1 copy of effect allele.

 © 2024. The Author(s).

Supplementary Table S5 Bidirectional causal association between myeloproliferative neoplasm and C-reactive protein using Mendelian randomization

Inverse variance weighting								MR-Egger					Weighted median		
Exposure	Outcome	SNP	OR/Beta	95% CI	*p*-Val	Q	Q *p*-val	OR/Beta	CI	*p*-Val	Intercept	Intercept *p*-val	OR/Beta	95% CI	*p*-Val
CRP[1]	MPN	49	1.314	0.941–1.835	0.109	63.673	0.064	1.833	1.147–2.929	0.015	−0.028	0.059	1.349	0.867–2.101	0.185
CRP[2]	MPN	67	1.298	0.923–1.828	0.134	94.866	0.011	1.789	1.111–2.879	0.020	−0.024	0.068	1.353	0.879–2.083	0.169
MPN[1]	CRP	6	-0.0005	−0.0091–0.0081	0.9077	3.0514	0.6921	0.0007	−0.0203–0.0217	0.9525	−0.0005	0.9090	−0.0002	−0.0101–0.0096	0.9650
MPN[2]	CRP	10	0.001	−0.006–0.008	0.773	3.918	0.917	−0.0005	−0.018–0.017	0.959	0.0007	0.852	0.0009	−0.009–0.0103	0.855

Abbreviations: CI, confidence interval; CRP, C-reactive protein; MPN, myeloproliferative neoplasm; OR, odds ratio; *p*-val, *p*-value; Q, Cochran Q statistics; SNPs, single nucleotide polymorphisms.
Note: CRP[1] indicates that the SNPs are associated with exposure at a genome-wide significance level. CRP[2] indicates that the SNPs are associated with exposure at $p < 5 \times 10^{-7}$. MPN[1] indicates that the SNPs are associated with exposure at $p < 5 \times 10^{-6}$. MPN[2] indicates that the SNPs are associated with exposure at $p < 1 \times 10^{-5}$. OR and 95% CI represent change in odds ratio of myeloproliferative neoplasm per 1 percent increase in CRP level. Beta and 95% CI represent change in percent of CRP per log odds increase in myeloproliferative neoplasm.

 © 2024. The Author(s).

Supplementary Table S6 Details of systemic inflammatory regulators predicting SNPs with myeloproliferative neoplasm (with genome-wide significant SNPs)

Systemic inflammatory regulators						Myeloproliferative neoplasm		*F*-statistics
SNP	Effect allele	Other allele	*p*-Value	Beta	Standard error	Beta	Standard error	
MIP1b								
rs113010081	C	T	3.85E-140	0.595	0.024	−0.044275571	0.0843	636.4930336
rs117453826	G	A	5.07E-22	0.577	0.059	0.109739461	0.1887	94.80782257
rs2079664	G	A	1.51E-08	−0.100	0.018	0.16848007	0.0635	31.96103435
Eotaxin								
rs112347425	T	C	8.65E-09	0.158	0.0277	−0.095654734	0.0999	32.53528653
rs12075	A	G	1.33E-26	0.1671	0.0156	−0.024950491	0.0567	114.7370562
rs2024050	G	A	1.10E-08	−0.1728	0.0303	0.363420035	0.1098	32.52387021
rs2228467	C	T	2.27E-46	0.4163	0.0292	0.003964254	0.1062	203.2577524
MCP1								
rs12075	A	G	1.44E-44	0.2185	0.0155	−0.019081131	0.0567	198.7190427
rs2036297	G	A	1.09E-13	−0.119	0.016	−0.158024799	0.0583	55.31640625
rs2288370	C	T	2.25E-10	0.1031	0.0163	0.400174353	0.0585	40.00756521
rs7632755	A	G	1.18E-20	0.2938	0.0316	−0.001478198	0.1081	86.4429178
SCGFb								
rs116924815	T	C	1.74E-16	0.6079	0.0738	0.118807653	0.1736	67.85026733
rs117716477	A	C	1.34E-23	0.8384	0.0841	−0.019321546	0.2311	99.38264424
rs17876031	G	A	2.25E-09	0.1514	0.0255	0.111872423	0.0606	35.25099577
rs4656185	A	G	1.16E-15	0.205	0.0256	0.148295677	0.0613	64.12506104
PDGFbb								
rs13412535	A	G	2.46E-55	0.3352	0.0214	−0.098079034	0.0713	245.3468425
rs2324229	C	T	3.48E-08	−0.0894	0.0161	−0.096671814	0.0586	30.83353266
rs4965869	T	C	5.66E-24	0.184	0.0181	0.215495034	0.0656	103.3423888
rs55680718	T	C	1.86E-08	−0.1383	0.0246	−0.388133029	0.0865	31.60633551
IL − 16								
rs1801020	G	A	4.53E-10	−0.1733	0.0272	−0.040096432	0.0644	40.59376352
rs4253283	C	T	1.75E-08	−0.146	0.0262	−0.045511682	0.0614	31.05296894
rs4778636	A	G	1.11E-30	−0.7272	0.0633	0.076861523	0.1354	131.9776285

Note: F-statistic >10 suggests sufficient strength to ensure the validity of the instrumental variable. Beta for systemic inflammatory regulators represents change in standard deviation per 1 copy of effect allele.

 © 2024. The Author(s).

Supplementary Table S7 Association of systemic inflammatory regulators with myeloproliferative neoplasm using Mendelian randomization (with genome-wide significant SNPs)

Inverse variance weighting								MR-Egger					Weighted median		
Category	Exposure	SNP	OR/ Beta	95% CI	*p*-Val	Q	Q *p*-val	OR/ Beta	95% CI	*p*-Val	intercept	Intercept *p*-Val	OR/ Beta	95% CI	*p*-Val
Chemokines															
	MIP1b	3	0.972	0.757–1.247	0.822	1.435	0.488	0.873	0.587–1.299	0.625	0.053	0.621	0.969	0.744–1.262	0.815
	Eotaxin	4	1.041	0.723–1.498	0.829	1.839	0.606	0.993	0.403–2.449	0.990	0.012	0.921	0.985	0.649–1.494	0.943
	MCP1	4	1.023	0.705–1.485	0.905	3.202	0.361	0.733	0.237–2.268	0.644	0.062	0.597	0.968	0.636–1.473	0.879
Growth factors															
	SCGFb	4	1.211	0.897–1.633	0.211	1.094	0.778	1.045	0.613–1.782	0.886	0.048	0.581	1.305	0.929–1.832	0.124
	PDGFbb	4	0.894	0.588–1.360	0.602	4.787	0.188	0.870	0.285–2.659	0.830	0.006	0.962	0.799	0.549–1.162	0.240
Interleukins															
	IL – 16	3	1.096	0.810–1.485	0.552	0.673	0.714	1.287	0.788–2.101	0.497	-0.056	0.564	1.084	0.788–1.490	0.621

Abbreviations: CI, confidence interval; OR, odds ratio; *p*-val, *p*-value; Q, Cochran Q statistics; SNPs, single nucleotide polymorphisms.
Note: OR and 95% CI represent change in odds ratio of myeloproliferative neoplasm per 1 SD increase in systemic inflammatory regulators level. After correcting for multiple comparison, *p*-value $< 0.05/6 = 0.0083$ was considered as significant.

 © 2024. The Author(s).

Supplementary Table S8 Details of systemic inflammatory regulators predicting SNPs with myeloproliferative neoplasm (with SNPs reaching $p < 5 \times 10^{-6}$)

►Table S8 displayed in Table S8.xlsx.

Supplementary Table S9 Association of systemic inflammatory regulators with myeloproliferative neoplasm using Mendelian randomization (with SNPs reaching $p < 5 \times 10^{-6}$)

Inverse variance weighting								MR-Egger					Weighted median		
Category	Exposure	SNP	OR/Beta	95%CI	*p*-val	Q	Q *p*-val	OR/Beta	95%CI	*p*-val	intercept	Intercept *p*-val	OR/Beta	95%CI	*p*-val
Chemokines															
	MIP1b	26	0.976	0.829–1.148	0.767	18.894	0.802	0.949	0.724–1.244	0.707	0.007	0.803	0.922	0.731–1.162	0.489
	Eotaxin	18	1.161	0.896–1.505	0.258	11.481	0.830	1.048	0.566–1.941	0.883	0.015	0.724	1.010	0.691–1.477	0.959
	MCP1	13	1.015	0.754–1.366	0.924	8.222	0.768	0.881	0.449–1.730	0.721	0.021	0.658	0.972	0.643–1.470	0.894
	MIG	9	1.275	0.952–1.708	0.103	8.095	0.424	0.957	0.454–2.018	0.912	0.065	0.438	1.298	0.876–1.923	0.193
	IP–10	8	1.336	0.955–1.868	0.091	6.895	0.440	1.238	0.558–2.746	0.618	0.013	0.841	1.361	0.884–2.095	0.162
	CTACK	11	1.024	0.810–1.296	0.840	5.388	0.864	0.877	0.534–1.441	0.617	0.040	0.504	1.076	0.785–1.477	0.648
	RANTES	8	0.896	0.645–1.244	0.511	5.395	0.612	0.568	0.236–1.368	0.254	0.101	0.3155	0.815	0.529–1.255	0.353
	MIP1a	7	1.343	0.862–2.094	0.192	8.984	0.174	1.196	0.320–4.470	0.801	0.022	0.860	1.342	0.788–2.285	0.279
	GROa	7	0.953	0.785–1.158	0.630	5.362	0.498	1.045	0.668–1.635	0.855	−0.0315	0.674	0.999	0.796–1.255	0.997
	SDF1a	6	1.132	0.458–2.796	0.788	8.078	0.152	2.073	0.046–93.681	0.727	−0.053	0.763	1.187	0.465–3.031	0.720
	MCP3	3	1.058	0.776–1.442	0.721	1.621	0.445	0.578	0.207–1.616	0.486	0.184	0.440	1.070	0.717–1.597	0.740
Growth factors															
	SCGFb	10	1.076	0.846–1.368	0.550	6.562	0.683	1.125	0.722–1.754	0.616	−0.011	0.820	1.137	0.833–1.553	0.419
	PDGFbb	13	0.978	0.750–1.274	0.867	10.873	0.540	0.913	0.534–1.559	0.744	0.011	0.777	0.799	0.554–1.152	0.230
	SCF	9	1.369	0.886–2.116	0.157	7.406	0.493	0.943	0.342–2.610	0.913	0.051	0.451	1.732	0.976–3.071	0.060
	GCSF	4	1.090	0.581–2.044	0.789	2.732	0.435	0.782	0.253–2.414	0.711	0.046	0.551	0.860	0.402–1.840	0.697
	VEGF	16	0.848	0.710–1.012	0.067	7.973	0.925	0.901	0.658–1.233	0.524	−0.015	0.653	0.909	0.737–1.122	0.374
	HGF	6	0.707	0.330–1.513	0.372	11.344	0.045	0.509	0.077–3.359	0.521	0.058	0.723	0.747	0.372–1.500	0.412
	MCSF	3	0.784	0.467–1.319	0.360	3.182	0.204	1.492	0.652–3.413	0.517	−0.171	0.320	0.860	0.511–1.448	0.571
	bNGF	7	1.271	0.872–1.853	0.211	6.955	0.325	5.098	0.918–28.322	0.122	−0.209	0.166	1.067	0.654–1.743	0.794
	FGFBasic	4	1.397	0.603–3.238	0.435	4.526	0.210	6.384	0.063–642.928	0.513	−0.155	0.578	1.392	0.589–3.291	0.451

 © 2024. The Author(s).

Supplementary Table S9 (*Continued*)

Inverse variance weighting								MR-Egger					Weighted median		
Category	Exposure	SNP	OR/Beta	95%CI	*p*-val	Q	Q *p*-val	OR/Beta	95%CI	*p*-val	intercept	Intercept *p*-val	OR/Beta	95%CI	*p*-val
Interleukins															
	IL-18	12	1.145	0.922–1.423	0.221	7.024	0.797	1.211	0.754–1.944	0.447	−0.013	0.801	1.320	0.976–1.783	0.071
	1L-12p70	10	0.887	0.692–1.136	0.343	7.810	0.553	0.849	0.561–1.285	0.460	0.010	0.801	0.857	0.647–1.135	0.282
	IL-10	11	0.977	0.733–1.303	0.875	4.719	0.909	0.678	0.370–1.244	0.241	0.054	0.213	0.821	0.572–1.179	0.286
	IL-17	4	0.798	0.421–1.511	0.488	1.573	0.666	1.249	0.108–14.453	0.875	−0.056	0.745	0.739	0.346–1.580	0.436
	IL-7	4	0.900	0.633–1.281	0.560	4.411	0.220	0.671	0.184–2.448	0.608	0.075	0.685	0.881	0.646–1.202	0.423
	IL-16	9	0.988	0.780–1.252	0.922	4.347	0.825	1.170	0.774–1.768	0.481	−0.051	0.362	1.005	0.743–1.359	0.975
	IL-2	8	1.128	0.809–1.572	0.479	8.511	0.290	1.528	0.783–2.981	0.260	−0.058	0.344	1.385	0.907–2.116	0.132
	IL-4	6	1.044	0.599–1.820	0.879	2.965	0.705	0.964	0.346–2.688	0.948	0.014	0.865	0.990	0.490–1.999	0.977
	IL-13	10	0.936	0.711–1.232	0.638	16.471	0.058	0.791	0.458–1.365	0.424	0.046	0.499	0.874	0.687–1.113	0.275
	IL1ra	6	1.161	0.782–1.724	0.460	3.543	0.617	1.491	0.546–4.067	0.479	−0.042	0.624	1.377	0.845–2.244	0.198
	IL2ra	5	1.377	1.006–1.883	0.046	4.932	0.294	1.057	0.677–1.649	0.824	0.099	0.229	1.290	0.930–1.788	0.127
	IL-6	4	1.149	0.556–2.373	0.707	3.411	0.333	0.433	0.106–1.762	0.363	0.136	0.259	1.299	0.574–2.941	0.530
	IL-9	4	0.870	0.463–1.636	0.666	4.175	0.243	0.371	0.090–1.527	0.303	0.177	0.326	0.846	0.400–1.790	0.662
	IL-1b	6	1.205	0.722–2.011	0.476	6.783	0.237	0.622	0.219–1.767	0.423	0.101	0.237	1.237	0.704–2.173	0.459
	IL-5	4	0.723	0.471–1.109	0.137	0.775	0.855	0.550	0.204–1.484	0.359	0.053	0.610	0.688	0.414–1.145	0.150
	IL-8	4	1.323	0.823–2.125	0.247	1.691	0.639	1.018	0.304–3.405	0.979	0.040	0.689	1.243	0.694–2.225	0.465
Other															
	TRAIL	18	0.989	0.793–1.235	0.924	15.706	0.545	0.945	0.685–1.304	0.736	0.0112	0.706	0.990	0.719–1.364	0.953
	IFNg	5	1.476	0.780–2.796	0.232	0.479	0.976	0.945	0.158–5.645	0.954	0.049	0.636	1.336	0.607–2.939	0.471
	MIF	5	0.641	0.427–0.964	0.032	1.966	0.742	0.747	0.367–1.521	0.480	−0.045	0.645	0.635	0.369–1.094	0.102
	TNFa	3	1.594	0.916–2.774	0.099	0.063	0.969	1.503	0.437–5.173	0.635	0.010	0.934	1.539	0.795–2.977	0.201
	TNFb	4	0.796	0.576–1.098	0.165	0.601	0.896	0.923	0.334–2.546	0.891	−0.035	0.791	0.775	0.532–1.130	0.185

Abbreviations: CI, confidence interval; OR, odds ratio; *p*-val, *p*-value; Q, Cochran Q statistics; SNPs, single nucleotide polymorphisms.
Note: OR and 95% CI represent change in odds ratio of myeloproliferative neoplasm per 1 SD increase in systemic inflammatory regulators level. After correcting for multiple comparison, p-value $< 0.05/41 = 0.0012$ was considered as significant.

 © 2024. The Author(s).

Supplementary Table S10. Details of myeloproliferative neoplasm predicting SNPs with systemic inflammatory regulators (with SNPs reaching $P < 5 \times 10^{-6}$)

►Table S10 displayed in Table S10.xlsx

Supplementary Table S11 Association of myeloproliferative neoplasm with systemic inflammatory regulators using Mendelian randomization. (with SNPs reaching $p < 5 \times 10^{-6}$)

Inverse variance weighting								MR-Egger					Weighted Median		
Category	Outcome	SNP	OR/ Beta	95%CI	*p*-val	Q	Q *p*-val	OR/ Beta	95%CI	*p*-val	intercept	Intercept *p*-val	OR/ Beta	95%CI	*p*-val
Chemokines															
	MIP1b	8	0.005	−0.031–0.041	0.794	9.904	0.194	−0.018	−0.128–0.093	0.764	0.009	0.685	−0.001	−0.041–0.038	0.948
	Eotaxin	8	−0.009	−0.048–0.030	0.666	11.449	0.120	0.0826	−0.011–0.176	0.133	−0.038	0.087	0.016	−0.024–0.056	0.440
	MCP1	8	0.007	−0.024–0.037	0.670	4.590	0.710	−0.021	−0.108–0.066	0.987	0.012	0.534	0.0003	−0.039–0.040	0.987
	MIG	8	0.0265	−0.0573–0.0839	0.3647	10.237	0.1755	0.043	−0.127–0.214	0.638	−0.005	0.879	0.051	−0.010–0.111	0.101
	IP-10	8	−0.00068	−0.046–0.045	0.977	4.753	0.690	0.090	−0.041–0.222	0.227	−0.038	0.199	0.026	−0.033–0.086	0.381
	CTACK	8	−0.0065	−0.052–0.0394	0.781	5.799	0.563	0.038	−0.095–0.170	0.597	−0.019	0.511	0.0070	−0.054–0.068	0.822
	RANTES	8	0.047	−0.0002–0.094	0.0514	2.796	0.903	0.051	−0.085–0.187	0.490	−0.0017	0.953	0.042	−0.017–0.102	0.163
	MIP1a	8	−0.0335	−0.1043–0.0373	0.3539	16.0894	0.0243	0.0279	−0.186–0.242	0.806	−0.026	0.570	−0.0414	−0.106–0.023	0.206
	GROa	8	−0.013	−0.077–0.052	0.701	13.387	0.063	0.041	−0.154–0.237	0.695	−0.022	0.587	−0.008	−0.069–0.054	0.807
	SDF1a	8	0.003	−0.033–0.039	0.860	9.199	0.239	−0.031	−0.139–0.076	0.587	0.015	0.526	−0.012	−0.054–0.030	0.574
	MCP3	8	0.070	−0.014–0.155	0.1039	3.107	0.875	0.107	−0.138–0.352	0.426	−0.015	0.766	0.071	−0.035–0.178	0.190
Growth factors															
	SCGFb	8	0.027	−0.019–0.072	0.254	4.345	0.739	−0.051	−0.182–0.081	0.478	0.032	0.265	0.009	−0.052–0.069	0.779
	PDGFbb	8	0	−0.040–0.040	0.998	12.224	0.0934	−0.0131	−0.137–0.111	0.843	0.0055	0.832	−0.0151	−0.056–0.0259	0.470
	SCF	8	0.0017	−0.029–0.033	0.916	7.255	0.4028	−0.0716	−0.159–0.016	0.160	0.0307	0.130	−0.0129	−0.053–0.0270	0.526
	GCSF	8	0.0172	−0.0138–0.0482	0.2773	4.054	0.774	−0.0075	−0.097–0.0816	0.8746	0.0104	0.5836	0.0033	−0.0359–0.0424	0.8703
	VEGF	8	0.0063	−0.027–0.039	0.708	3.823	0.800	0.026	−0.069–0.120	0.612	−0.008	0.682	0.009	−0.0321–0.051	0.660
	HGF	8	0.0251	−0.0053–0.055	0.105	5.776	0.567	−0.023	−0.110–0.065	0.628	0.020	0.296	0.014	−0.024–0.052	0.470
	MCSF	8	−0.008	−0.073–0.058	0.817	9.684	0.207	−0.039	−0.241–0.163	0.718	0.013	0.757	−0.034	−0.107–0.039	0.363
	bNGF	8	−0.036	−0.083–0.011	0.132	1.763	0.972	−0.092	−0.226–0.042	0.229	0.023	0.417	−0.044	−0.101–0.013	0.132
	FGFBasic	8	−0.003	−0.039–0.033	0.865	8.972	0.255	0.010	−0.101–0.120	0.871	−0.005	0.819	−0.015	−0.057–0.028	0.501

 © 2024. The Author(s).

Supplementary Table S11 (*Continued*)

Inverse variance weighting								MR-Egger					Weighted Median		
Category	Outcome	SNP	OR/ Beta	95%CI	*p*-val	Q	Q *p*-val	OR/ Beta	95%CI	*p*-val	intercept	Intercept *p*-val	OR/ Beta	95%CI	*p*-val
Interleukins															
	IL-18	8	0.011	−0.035–0.057	0.635	4.196	0.757	−0.039	−0.171–0.093	0.586	0.021	0.460	−0.0005	−0.059–0.058	0.985
	IL-12p70	8	0.019	−0.012–0.049	0.226	3.319	0.854	0.003	−0.084–0.091	0.945	0.007	0.723	0.009	−0.028–0.046	0.637
	IL-10	8	0.018	−0.013–0.050	0.251	6.120	0.526	0.070	−0.020–0.161	0.179	−0.022	0.276	0.023	−0.018–0.064	0.280
	IL-17	8	0.009	−0.028–0.045	0.638	9.456	0.222	−0.003	−0.115–0.110	0.966	0.005	0.841	−0.005	−0.047–0.038	0.834
	IL-7	8	0.027	−0.026–0.079	0.317	8.589	0.284	0.131	−0.006–0.267	0.109	−0.044	0.162	0.041	−0.020–0.103	0.187
	IL-16	8	0.026	−0.021–0.0727	0.280	1.773	0.971	−0.005	−0.140–0.130	0.945	0.013	0.650	0.005	−0.052–0.062	0.865
	IL-2	8	−0.007	−0.054–0.040	0.767	5.617	0.585	0.063	−0.072–0.198	0.399	−0.029	0.322	−0.003	−0.064–0.057	0.918
	IL-4	8	−0.012	−0.043–0.019	0.447	1.615	0.978	−0.020	−0.108–0.069	0.679	0.003	0.861	−0.014	−0.054 – 0.026	0.492
	IL-13	8	−0.007	−0.066–0.052	0.819	11.185	0.131	0.015	−0.167–0.197	0.875	−0.009	0.808	−0.016	−0.079–0.047	0.619
	IL1ra	8	−0.037	−0.096–0.022	0.222	11.577	0.115	0.015	−0.163–0.193	0.878	−0.021	0.569	−0.045	−0.107–0.016	0.149
	IL2ra	8	−0.0016	−0.047–0.044	0.946	5.764	0.568	0.004	−0.128–0.135	0.957	−0.002	0.935	0.0035	−0.056–0.063	0.907
	IL-6	8	0.00016	−0.030–0.031	0.992	3.869	0.795	0.003	−0.085–0.091	0.951	−0.001	0.951	−0.005	−0.042 – 0.032	0.786
	IL-9	8	−0.021	−0.067–0.025	0.380	6.057	0.533	−0.064	−0.196–0.068	0.379	0.018	0.518	−0.041	−0.101–0.019	0.184
	IL-1b	8	−0.019	−0.060–0.021	0.348	8.600	0.283	0.022	−0.099–0.143	0.736	−0.017	0.503	−0.017	−0.066–0.031	0.486
	IL-5	8	0.013	−0.035–0.074	0.590	5.979	0.542	0.077	−0.061–0.215	0.314	−0.027	0.369	0.008	−0.058–0.073	0.815
	IL-8	8	−0.017	−0.073–0.038	0.541	9.923	0.193	0.072	−0.083–0.227	0.399	−0.037	0.275	0.021	−0.042–0.084	0.507
Other															
	TRAIL	8	−0.019	−0.062–0.024	0.395	14.209	0.048	−0.004	−0.138–0.130	0.958	−0.006	0.822	−0.010	−0.049–0.029	0.606
	IFNg	8	0.0012	−0.041–0.043	0.957	12.433	0.087	0.016	−0.114–0.146	0.815	−0.006	0.817	−0.005	−0.045–0.036	0.813
	MIF	8	0.021	−0.028–0.069	0.405	7.585	0.371	−0.126	−0.261–0.009	0.117	0.061	0.064	−0.004	−0.066–0.0584	0.899
	TNFa	8	−0.020	−0.076–0.037	0.495	9.858	0.197	0.101	−0.039–0.242	0.206	−0.051	0.122	−0.011	−0.071–0.048	0.714
	TNFb	8	0.021	−0.049–0.091	0.553	5.735	0.571	−0.076	−0.277–0.125	0.488	0.041	0.351	−0.014	−0.106–0.078	0.763

Abbreviations: CI, confidence interval; *p*-val, *p*-value; Q, Cochran Q statistics; SNPs, single nucleotide polymorphisms.
Note: Beta and 95% CI represent change in SD of Inflammatory regulators per log odds increase in myeloproliferative neoplasm. After correcting for multiple comparison, *p*-value $< 0.05/41 = 0.0012$ was considered as significant.

 © 2024. The Author(s).

Supplementary Table S12 Details of myeloproliferative neoplasm predicting SNPs with systemic inflammatory regulators (with SNPs reaching $p < 1 \times 10^{-5}$).

►**Table S12 displayed in Table S12.xlsx**

Supplementary Table S13 Association of myeloproliferative neoplasm with systemic inflammatory regulators using Mendelian randomization (with SNPs reaching $p < 1 \times 10^{-5}$)

Inverse variance weighting								MR-Egger					Weighted median		
Category	Outcome	SNP	OR/Beta	95%CI	*p*-val	Q	Q *p*-val	OR/Beta	95%CI	*p*-val	intercept	Intercept *p*-val	OR/Beta	95%CI	*p*-val
Chemokines															
	MIP1b	14	0.0049	−0.0237–0.0335	0.736	15.902	0.254	−0.0303	−0.1094–0.0488	0.468	0.014	0.368	0.0076	−0.0281–0.0432	0.677
	Eotaxin	14	−0.0047	−0.0385–0.029	0.784	21.948	0.056	0.0639	−0.0236–0.1514	0.178	−0.027	0.125	0.0159	−0.0222–0.0541	0.412
	MCP1	14	0.0014	−0.0244–0.0273	0.914	8.388	0.817	−0.0096	−0.0809–0.0616	0.795	0.004	0.749	0.0006	−0.0361–0.0372	0.976
	MIG	14	0.0059	−0.0364–0.0481	0.785	15.572	0.273	0.0434	−0.0755–0.1622	0.488	−0.015	0.519	0.0468	−0.0102–0.1038	0.107
	IP-10	14	−0.0042	−0.0429–0.0345	0.831	10.888	0.620	0.0415	−0.0651–0.1481	0.460	−0.018	0.385	0.0115	−0.0415–0.0644	0.671
	CTACK	14	−0.0076	−0.0466–0.0314	0.703	11.040	0.608	0.0599	−0.0477–0.1675	0.297	−0.027	0.212	0.0055	−0.0511–0.0622	0.849
	RANTES	13	0.0432	0.0021–0.0843	0.039	4.310	0.977	0.0509	−0.0598–0.1615	0.387	−0.003	0.887	0.0449	−0.0119–0.1017	0.121
	MIP1a	14	−0.021	−0.074–0.032	0.438	23.230	0.039	−0.0088	−0.1603–0.1426	0.911	−0.005	0.869	−0.0325	−0.0898–0.0248	0.266
	GROa	14	−0.0094	−0.0538–0.035	0.677	16.287	0.234	0.0165	−0.1101–0.143	0.803	−0.010	0.675	−0.0065	−0.064–0.051	0.825
	SDF1a	14	0.0085	−0.0256–0.0427	0.624	21.398	0.065	−0.0319	−0.1269–0.0631	0.523	0.016	0.388	−0.0182	−0.0575–0.0211	0.364
	MCP3	13	0.0463	−0.0273–0.1198	0.218	9.792	0.634	0.0412	−0.1782–0.2605	0.720	0.002	0.962	0.0623	−0.0377–0.1622	0.222
Growth factors															
	SCGFb	14	0.0049	−0.0363–0.046	0.816	14.675	0.328	−0.0903	−0.1968–0.0163	0.123	0.038	0.085	0.0095	−0.0476–0.0667	0.743
	PDGFbb	14	0.0118	−0.0218–0.0453	0.492	21.971	0.056	0.0006	−0.0955–0.0967	0.990	0.004	0.811	−0.0123	−0.0495–0.025	0.519
	SCF	14	−0.0014	−0.0272–0.0244	0.914	10.823	0.626	−0.0727	−0.1439–0.0016	0.068	0.029	0.057	−0.0128	−0.0509–0.0252	0.509
	GCSF	14	0.0167	−0.0097–0.0431	0.214	10.648	0.640	0.0136	−0.0589–0.0862	0.719	0.001	0.931	0.0033	−0.0332–0.0397	0.861
	VEGF	14	0.0094	−0.0202–0.0391	0.532	14.610	0.332	0.0124	−0.0725–0.0973	0.780	−0.001	0.943	0.0094	−0.0306–0.0494	0.646
	HGF	14	0.0148	−0.011–0.0407	0.261	13.056	0.443	0.028	−0.0458–0.1018	0.472	−0.005	0.715	0.0067	−0.0302–0.0437	0.721
	MCSF	14	−0.0271	−0.0819–0.0278	0.334	17.563	0.175	−0.0933	−0.2447–0.0582	0.251	0.026	0.376	−0.0354	−0.1051–0.0343	0.319
	bNGF	14	−0.028	−0.0676–0.0116	0.166	6.010	0.946	−0.0857	−0.1946 – 0.0231	0.149	0.023	0.286	−0.0419	−0.0937–0.0098	0.112
	FGFBasic	14	0.0032	−0.0264–0.0328	0.832	15.704	0.266	0.0136	−0.0709–0.0981	0.757	−0.004	0.799	−0.0181	−0.0578–0.0216	0.372

 © 2024. The Author(s).

Supplementary Table S13 (*Continued*)

Inverse variance weighting								MR-Egger					Weighted median		
Category	Outcome	SNP	OR/Beta	95%CI	*p*-val	Q	Q *p*-val	OR/Beta	95%CI	*p*-val	intercept	Intercept *p*-val	OR/Beta	95%CI	*p*-val
Interleukins															
	IL-18	14	0.015	−0.024–0.054	0.452	7.656	0.865	0.015	−0.0924–0.1223	0.789	0.000	1.000	0.0006	−0.0532–0.0544	0.982
	1L-12p70	14	0.0224	−0.0034–0.0483	0.089	12.933	0.453	0.0155	−0.0583–0.0893	0.687	0.003	0.847	0.0107	−0.0257–0.047	0.564
	IL-10	13	0.0304	0.0013–0.0595	0.041	13.549	0.330	0.0457	−0.0356–0.1271	0.294	−0.006	0.698	0.028	−0.0128–0.0687	0.179
	IL-17	14	0.0156	−0.017–0.0483	0.348	19.510	0.108	−0.003	−0.0957–0.0898	0.951	0.007	0.681	0.005	−0.0332–0.0432	0.797
	IL-7	14	0.0136	−0.0265–0.0538	0.506	12.954	0.451	0.0605	−0.0505–0.1716	0.306	−0.019	0.392	0.0276	−0.0286–0.0838	0.336
	IL-16	14	0.013	−0.0267–0.0528	0.521	7.578	0.870	0.0149	−0.0946–0.1245	0.794	−0.001	0.971	0.0037	−0.0528–0.0601	0.894
	IL-2	14	0.0094	−0.0379–0.0567	0.697	18.386	0.143	−0.0256	−0.1591–0.1079	0.714	0.014	0.591	0.0023	−0.0536–0.0581	0.939
	IL-4	14	0.0052	−0.0245–0.035	0.731	16.877	0.205	0.0117	−0.0736–0.097	0.793	−0.003	0.876	−0.0117	−0.0476–0.0242	0.531
	IL-13	14	−0.0096	−0.0559–0.0367	0.685	17.951	0.159	−0.0253	−0.1577–0.1071	0.715	0.006	0.808	−0.0093	−0.0667–0.0481	0.751
	IL1ra	14	−0.0124	−0.0623 – 0.0375	0.626	21.350	0.066	−0.0247	−0.1672–0.1178	0.740	0.005	0.859	−0.0337	−0.0893–0.0219	0.235
	IL2ra	14	0.0129	−0.0258–0.0517	0.513	9.460	0.737	0.0169	−0.0897–0.1235	0.761	−0.002	0.938	0.0196	−0.0338–0.0731	0.472
	IL-6	14	0.0149	−0.0147–0.0446	0.323	16.867	0.205	−0.0082	−0.092–0.0756	0.851	0.009	0.572	−0.0031	−0.0415–0.0354	0.876
	IL-9	14	−0.0112	−0.0522–0.0299	0.594	14.365	0.349	−0.0495	−0.1646–0.0656	0.415	0.015	0.496	−0.0269	−0.0837–0.0298	0.352
	IL-1b	14	−0.0191	−0.0545–0.0163	0.290	17.007	0.199	−0.028	−0.1293–0.0734	0.598	0.004	0.857	−0.0146	−0.0605–0.0314	0.535
	IL-5	14	0.0173	−0.0235–0.0581	0.405	13.123	0.438	0.0238	−0.0928–0.1404	0.696	−0.003	0.909	0.0083	−0.0497–0.0664	0.778
	IL-8	14	0.0137	−0.0386–0.0659	0.608	22.645	0.046	0.0155	−0.1342–0.1651	0.843	−0.001	0.980	0.0223	−0.0366–0.0812	0.458
Other															
	TRAIL	14	−0.0149	−0.0485–0.0187	0.383	21.948	0.056	−0.0161	−0.1125–0.0803	0.749	0.000	0.981	−0.0102	−0.046–0.0257	0.579
	IFNg	14	0.0053	−0.0287–0.0392	0.760	20.929	0.074	0.0352	−0.0604–0.1307	0.485	−0.012	0.523	−0.0046	−0.0449–0.0357	0.824
	MIF	14	0.0054	−0.0343–0.0451	0.790	10.427	0.659	−0.0767	−0.186–0.0327	0.195	0.033	0.140	0.002	−0.0538–0.0577	0.945
	TNFa	14	−0.017	−0.0599–0.0258	0.436	14.872	0.315	0.0292	−0.0903–0.1487	0.641	−0.018	0.432	−0.0076	−0.0669–0.0516	0.801
	TNFb	14	0.0289	−0.0551–0.1129	0.500	22.490	0.032	−0.1783	−0.4014–0.0447	0.145	0.080	0.079	−0.0034	−0.0887–0.0819	0.938

Abbreviations: CI, confidence interval; *p*-val, *p*-value; Q, Cochran Q statistics; SNPs, single nucleotide polymorphisms.
Note: Beta and 95% CI represent change in SD of Inflammatory regulators per log odds increase in myeloproliferative neoplasm. After correcting for multiple comparison, *p*-value $< 0.05/41 = 0.0012$ was considered as significant.

 © 2024. The Author(s).

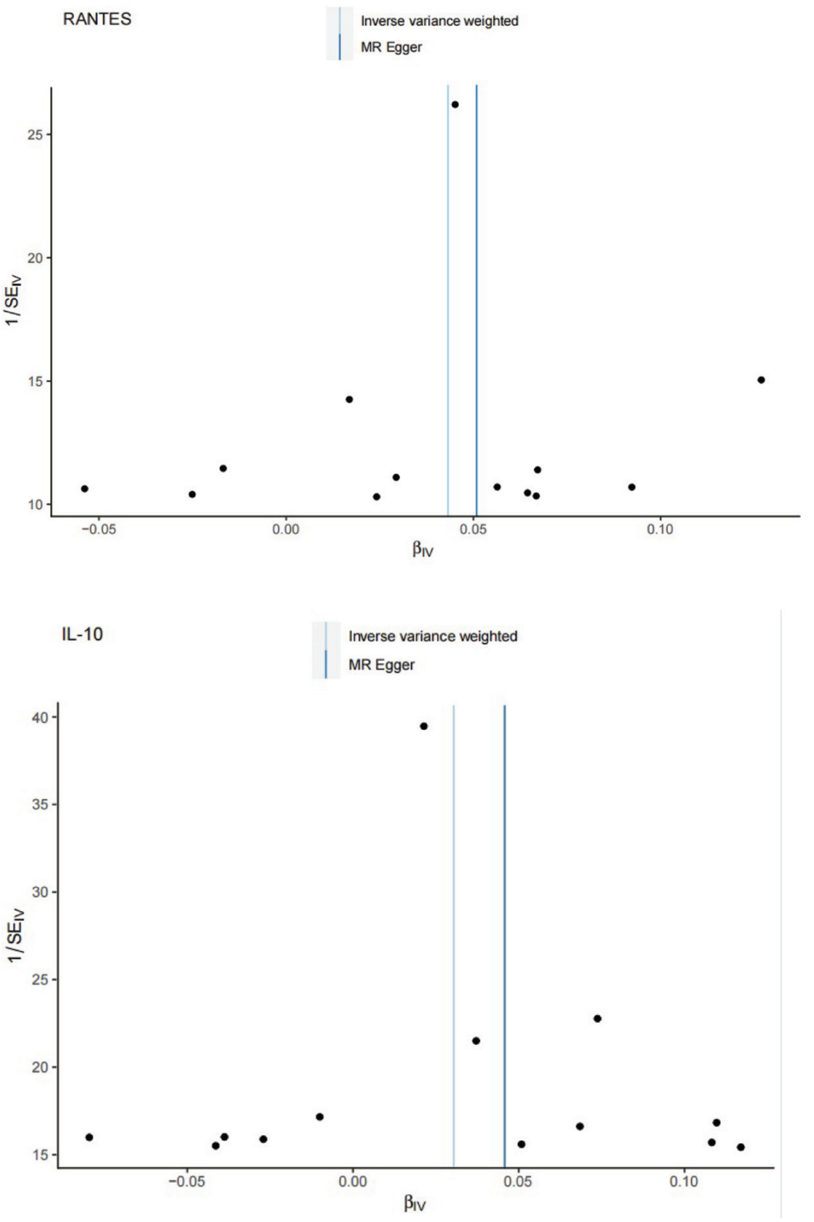

Supplementary Fig. S1 The Mendelian randomization (MR) funnel plots illustrating the relationship between myeloproliferative neoplasm (MPN) with RANTES and interleukin 10 (IL-10). The funnel plots show the inverse variance-weighted MR estimate of each MPN single-nucleotide polymorphism with cytokines versus 1/standard error (1/SEIV).

 © 2024. The Author(s).
